# Characteristics and outcomes of patients with an unscheduled return visit within 72 hours to the Paediatric Emergency Centre at a Private Tertiary Referral Hospital in Kenya

**DOI:** 10.1016/j.afjem.2021.03.003

**Published:** 2021-04-05

**Authors:** Kenneth M. Rintaari, Rachel Wangari Kimani, Horatius Malilu Musembi, Samwel Maina Gatimu

**Affiliations:** a School of Nursing and Midwifery, Aga Khan University, 00623 Nairobi, Kenya; b Accident and Emergency Department, Aga Khan University Hospital, 00100 Nairobi, Kenya; c School of Economics, University of Nairobi, 00100 Nairobi, Kenya

**Keywords:** Kenya, Unscheduled, Revisits, Emergency department, 72 hours

## Abstract

**Introduction:**

Patients’ unscheduled return visits (URVs) to the paediatric emergency Centre (PEC) contribute to overcrowding and affect health service delivery and overall quality of care. This study assessed the characteristics and outcomes of paediatric patients with URVs (within 72 hours) to the PEC at a private tertiary hospital in Kenya.

**Methods:**

We conducted a retrospective chart review of all URVs within 72 hours among paediatric patients aged ≤15 years between 1 July and 31 December 2018 at the tertiary hospital in Nairobi, Kenya.

**Results:**

During the study period, 1.6% (*n*=172) of patients who visited the PEC returned within 72 hours, with 4.7% revisiting the PEC more than once. Patients’ median age was 36 months (interquartile range: 42 months); over half were male (51.7%), 55.8% were ambulatory and 84.3% were insured. In addition, 21% (*n*=36) had chronic diseases and 7% (*n*=12) had drug allergies. Respiratory (59.5%) and gastrointestinal (21.5%) tract infections were the most common diagnoses. Compared with the first visit, more patients with URVs were classified as urgent (1.7% vs. 5.2%) and were non-ambulatory (44.2% vs. 49.5%, *p*=<0.001); 18% of these patients were admitted. Of these 58% were male, 83.9% were aged 0–5 years, 12.9% were classified as urgent, 64.5% had respiratory tract infections and 16.1% had gastrointestinal tract infections. Being admitted was associated with patient acuity (*p*=0.004), laboratory tests (*p*=<0.001) and ambulatory status (*p*=0.041).

**Conclusion:**

The URV rate is low in our setting. Patients who returned to the PEC within 72 hours tended to be male, under 5 years old and insured. Many were non-urgent cases with diagnoses of respiratory and gastrointestinal tract infections. The findings suggest that some URVs were necessary and may have contributed to better care and improved outcomes while others highlight a need for effective patient education and comprehensive initial assessment.

## African relevance

• Most emergency centres in Africa are congested and overcrowded and have limited financial and human resources for health.
• Paediatric patients return to emergency centres within a brief time after a previous visit contributing to the overcrowding.
• There is a lack of evidence on the burden of overcrowding and patients’ unscheduled return visits.
• In most countries in Africa, emergency centres need interventions to optimise care, decrease preventable causes of revisits, reduce healthcare costs and unnecessary emergency centre use, and ensure prompt care for urgent cases.

## Introduction

Patient revisits to emergency centres (ECs) increase congestion and overcrowding [1], resulting in increased pressure in ECs and workload for healthcare professionals [2]. Besides, waiting time and length of stay are increased [3], and emergency care delivery [4] and the overall quality of care [5] are impacted. However, unscheduled patient revisits to the EC could also highlight deficiencies in the initial patient assessment, treatment or intervention [[6], [7], [8]], and reveal inadequacies in hospital systems or healthcare providers [9]. Unscheduled return visits (URV) could be attributable to disease-related factors, including the natural progression of the disease [10], advance to acute and chronic illnesses [11] and the use of assistive devices [10]. Also, shortcomings in hospital systems (e.g. insufficient care provided during the initial visit and partial or untimely discharge [11]) and drug-related reasons (e.g. side effects of drugs, non-compliance and the use of wrong or substandard drugs [12]) could result in URVs. URVs, therefore, offer a useful indicator of the quality of care and patient safety [4,5].

The rate of URVs within 72 hours of discharge from paediatric accident and emergency centres (PECs) varies globally. For example, the URV rate was reported as 0.8% in India [13], 3% in Lithuania [14], 4.3% in Hong Kong [15] and 5.2% in Canada [16]. However, in low- and middle-income countries including Kenya [17], there is limited evidence of the burden and impact associated with URVs, despite ECs in these settings experiencing high rates of patient congestion and increased mortality attributed to EC care [18].

Between 2003 and 2013, Kenya saw an almost 90% increase in outpatients’ visits to 122 per 100 sick people within four weeks, with 30.6% of all these visits being in private health facilities compared to 58% in public health facilities [19]. Besides, 26.2% of outpatients visits by children 0–14 years old were in private health facilities with an average of 7.6 and 2.9 outpatient visits per year for under five and 5–14 years-old children [19]. Respiratory system, malaria, diseases of the skin, diarrhoeal diseases and urinary tract infections are the top five diseases resulting in most outpatient visits in public health facilities with pneumonia and malaria contributing to a quarter of all hospital admissions in 2019 [20]. The utilisation of health services in Kenya increases with socioeconomic status; with the wealthy individuals using outpatient, inpatient and preventive care more than the low-income individuals especially in private health facilities [21]. These differences in utilisation as well as the significant increase in outpatients visits are unexplained. However, URVs have been shown to increase overcrowding in ECs [1], which may explain the increased outpatients' visits. Hence, we characterised patients with URVs to the PEC within 72 hours at a private tertiary hospital in Kenya to address the paucity of evidence on the burden of URVs. The findings could further the understanding of health-seeking behaviours of Kenyans and contribute to addressing the challenges at the ECs.

The promotive, preventive and curative health service delivery in Kenya is devolved to 47 semi-autonomous counties while the national government oversees the referral health services [22]. Private (for-profit, not-for-profit and faith-based) and public health facilities are divided into six levels of care – level 1 (community), 2 (dispensaries), 3 (health centres), 4 (primary level hospitals), 5 (secondary level hospitals) and 6 (referral hospital) [22]. Overall, 86% and 82% of private and public health facilities have sufficient capacity to provide general health services respectively [23], with child and adolescent preventive and curative care being the most available health services in both [23]. Moreover, only about a fifth of Kenyan have health insurance cover [24] with 88% and 12% covered with the national and private health insurance, respectively [19].

## Methods

### Study design and setting

We conducted a retrospective review of records for paediatric patients with URVs within 72 hours of the index visit to the PEC at a tertiary hospital in Nairobi, Kenya. The hospital is a private secondary-level university teaching hospital with a 280-bed capacity and an average of 85 PEC patient visits each day. It is located in a high socioeconomic setting where 49.2% of people are from middle-to-high income households and 48.9% of outpatient visits are in private health facilities [19].

### Sample

**Fig. 1:**
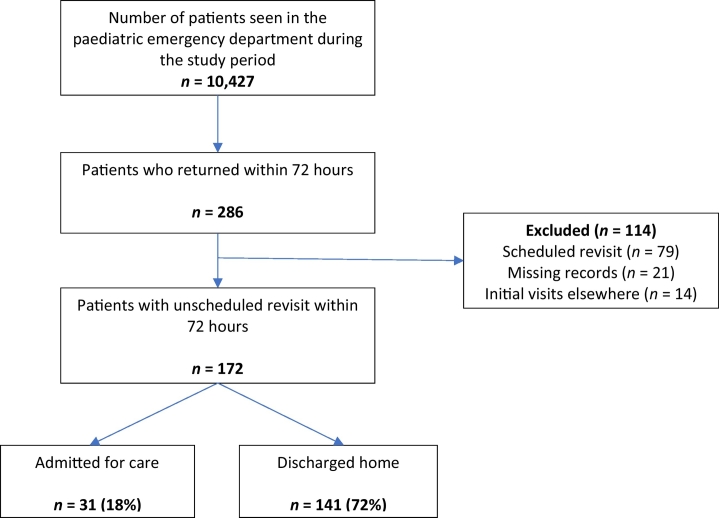
Flowchart of patient recruitment

The sample included all paediatric patients aged ≤15 years with URVs within 72 hours after the index visit seen at the PEC between 1 July and 31 December 2018. Revisiting patients with an index visit in a different facility, those revisiting after 72 hours from the first encounter, those with planned/scheduled revisits and patients aged >15 years were excluded (Fig. 1). Patients older than 15 years were excluded from this analysis because they are seen at the adult EC.

### Measures

Unscheduled revisit refers to any patient (≤15 years) returning to the PEC with the same chief complaint or a complaint related to the initial management within 72 hours of discharge. We retrieved data on patients’ demographic characteristics, visit time and date, mode of arrival to the PEC, acuity, pre-existing illnesses (if any), food and drug allergies (if any), prescribed medications, payment mode and the final disposition on revisits. Mode of arrival was classified as either ambulatory or non-ambulatory. Non-ambulatory patients were those who were carried or using a wheelchair, including critically ill infants and those with cerebral palsy. The patient’s acuity was assessed based on the Paediatric Canadian Triage and Acuity Scale (P–CTAS) criteria [25]. The scale classifies a patient into five levels according to their condition and the allowable length of waiting time. Level 1 patients require resuscitation and should be attended immediately, level 2 are emergent patients who should be seen immediately or ≤15 minutes while level 3 patients are referred as ‘urgent’ and should be seen in ≤ 30 minutes. Level 4 patients are semi-urgent and can be seen up to one hour while level 5 are non-urgent patients and can be seen up to two hours [25]. Patients were considered to have a pre-existing disease if they were already on treatment and follow-up for a particular disease but presented with complains other than the pre-existing disease. These data were collected from patients’ medical files using a checklist, and compared between the index visit and the revisit.

### Data analysis

We described patients’ characteristics for the index visit and revisit(s) using frequencies and percentages. Differences between the index visit and revisits and between patients who were discharged after revisit and those admitted for further management were assessed using chi-square and Fisher’s exact tests. All analyses were performed using STATA version 15, with the significance level set at 0.05.

### Ethics

This study's ethical approval was obtained from the University Research Ethics Committee (REF: 2019/REC-08(v1) and the Hospital Medical Records Department. Only de-identified data were retrieved from the medical files, and complete privacy and confidentiality was maintained through serialisation.

## Results

Of the 10,427 patients seen at the PEC during the study period, 172 patients had URVs within 72 hours (Fig. 1). All patients were triaged by a nurse and attended by a general practitioner. The prevalence rate of URVs was 1.64% (95% confidence interval: 1.41%–1.90%).

### Characteristics of patients with URV

Patients’ median age was 36 months (interquartile range: 42 months). Over half (51.7%) were male, 55.8% were ambulatory and most (84.3%) were insured. In addition, 21% (*n* = 36) had pre-existing diseases and 7% (*n* = 12) had drug allergies (Table 1). Respiratory (59.5%) and gastrointestinal (21.5%) tract infections were the most common reasons for URVs within 72 hours and admission after URV. Asthma (51.4%), eczema (22.9%) and cerebral palsy (8.6%) were the most prevalent pre-existing diseases among patients with URVs. All the children had up-to-date immunisation while none were HIV-positive.

**Table 1:** Sample characteristics.

Variables	*n* (%)
Gender	
Male	89 (51.7)
Female	83 (48.3)

Age, months	
Median (IQR)^a^	36 (12–54)
≤2	2 (1.2)
2–60	144 (83.7)
61–120	15 (8.7)
121–180	11 (6.4)

Mode of payment	
Cash	27 (15.7)
Insurance	145 (84.3)

Pre-existing diseases (*n* = 36)	
Asthma	18 (51.4)
Eczema	9 (22.9)
Cerebral palsy	3 (8.6)
Brain atrophy	1 (2.9)
Delayed speech	1 (2.9)
Rhinitis	1 (2.9)
Sinusitis	1 (2.9)
Congenital heart disease	1 (2.9)
Convulsive disorder	1 (2.9)

Drug and food allergies	
Yes	21 (12.2)
No	151 (87.8)

a IQR, interquartile range

### Comparisons of URVs and first visits

Compared with the first visit, more patients with URVs were classified as urgent (1.7% vs. 5.2%, *p* = 0.122) and non-ambulatory (44.2% vs. 49.5%, *p* = <0.001). Patients with URVs had more gastrointestinal tract cases, genitourinary and blood infections, and skin conditions than patients without URVs (Table 2).

**Table 2:** Comparison between first (index) visit and the unscheduled return visit.

Characteristics	Initial visit n (%)	Return visit n (%)	Percentage difference	*p*-value
Mode of arrival				
Non-ambulatory (carried/wheelchair)	76 (44.2)	84 (49.5)	5.3	<0.00^c^
Ambulatory	96 (55.8)	87 (50.6)	−5.2	

Acuity of the patient				
Urgent	3 (1.7)	9 (5.2)	3.5	
Semi-urgent	32 (18.6)	39 (22.7)	4.1	0.122
Non-urgent	137 (79.7)	124 (72.1)	−7.6	

Radiological investigations				
Yes^a^	8 (4.7)	11 (6.4)	1.7	0.030^c^
No	164 (95.3)	161 (93.6)	−1.7	

Laboratory tests				
Yes	25 (14.5)	41 (23.8)	9.3	>0.999
No	147 (85.5)	131 (76.2)	−9.3	

Type of laboratory tests	*n*=25	*n*=41		
Full blood count	13 (7.6)	41 (23.8)	16.2	
Urine	14 (8.1)	20 (11.6)	3.5	
Stool	5 (2.9)	21 (12.2)	9.3	
Urea, electrolytes, and creatinine	1 (6.0)	19 (11.0)	5.0	
Procalcitonin	4 (2.9)	15 (8.7)	5.8	
Others^b^	11 (6.4)	28 (16.3)	9.9	

Primary diagnosis				
Respiratory tract infections	104 (60.5)	103 (59.9)	−0.6	
Gastrointestinal tract infections	34 (19.8)	37 (21.5)	1.7	
Ear infections	15 (8.7)	8 (4.7)	−4.0	
Skin conditions	9 (5.2)	13 (7.6)	2.4	<0.001^c^
Central nervous system and musculoskeletal conditions	6 (3.5)	3 (2.3)	−1.2	
Eye infections	2 (1.2)	2 (1.2)	0.0	
Genitourinary infections	1 (0.6)	3 (1.7)	1.1	
Blood infections (neonatal sepsis and malaria)	1 (0.6)	3 (1.7)	1.1	

a X-ray, ultrasound, and computed tomography scan.

b Other laboratory tests (malaria (*n* = 2), C-reactive protein, blood culture and bone metabolism).

c Significant at *p* = 0.05

### Outcome after URVs

Thirty-one patients (18.0%) were admitted following their URV and 141 were discharged home. Among those who were admitted, 58% were male, 83.9% were aged 0–60 months, 12.9% were classified as urgent, 64.5% had respiratory tract infections and 16.1% had gastrointestinal tract infections. Besides, two patients with lactulose and sulphur drug allergies and eight patients with chronic diseases (asthma, *n* = 4; convulsive disorder, *n* = 2; brain atrophy, *n* = 1; congenital heart disease, *n* = 1) were admitted. There were significant associations between outcome and patient acuity (*p* = 0.004), laboratory tests (*p* = <0.001) and mode of arrival (*p* = 0.041) (Table 3).

**Table 3:** Outcomes after an unscheduled return visit

Characteristics	Admission n (%) N=31	Discharge n (%) N=141	*p*-value
Gender			
Male	18 (58.0)	71 (50.4)	0.437
Female	13 (42.0)	70 (49.6)	

Age, months			
0–60	26 (83.9)	120 (85.0)	
61–120	1 (3.2)	14 (10.0)	0.430
121–180	4 (13)	7 (5.0)	

Mode of payment			
Cash	2 (6.5)	25 (17.7)	0.418
Insurance	29 (93.5)	116 (82.3)	

Mode of arrival			
Non-ambulatory	21 (68.7)	64 (45.4)	0.041^a^
Ambulatory	10 (32.3)	77 (54.6)	

Acuity of the patient			
Urgent	4 (12.9)	5 (3.5)	
Semi-urgent	11 (35.5)	28 (19.9)	0.004^a^
Non-urgent	16 (51.6)	108 (76.6)	

Pre-existing diseases			
Yes	8 (25.8)	27 (19.2)	0.460
No	23 (74.2)	114 (80.8)	

Laboratory tests			
Yes	17 (54.8)	24 (17.0)	<0.001^a^
No	14 (45.2)	117 (83.0)	

Radiological investigations			
Yes	3 (6.5)	9 (6.4)	0.488
No	29 (93.5)	132 (93.6)	

Diagnosis			
Respiratory tract infections	20 (64.5)	82 (58.2)	
Gastrointestinal tract infections	5 (16.1)	32 (22.7)	
Central nervous system, skin, and musculoskeletal conditions	1 (3.2)	16 (11.3)	0.179
Ear infections	2 (6.5)	6 (4.3)	
Other infections^b^	3 (9.7)	5 (3.5)	

a Significant at *p* = 0.05.

b Other infections: eye, genitourinary and blood infections

## Discussion

Our study found that the rate of unscheduled return visits was 1.6%, which was twice the rate in India (0.8%) [13] but lower than in high-income countries (2.5%–5.2%) [7,[14], [15], [16],^26^,^27^]. Our study was conducted in a private tertiary and referral hospital with a small sample and unreliable paper-based medical files, which may explain the low URV rate. Also, patients in our study returned only once compared with other studies that patients had more than one URV within 72 hours [10]. However, the reported low URV rate could also reflect the quality of care provided at the study hospital.

Similar to other studies [15,28,29], most patients with URVs in our study were male and of young age. Toddlers and young children (younger than 3 years) tend to have frequent PEC visits and are associated with URVs [9,15,30]. Young children have poor communication ability, which makes it difficult to effectively report their symptoms and disease severity. Besides, their parents are likely to be anxious and may be inexperienced in dealing with a sick child (especially first-time parents), which may mean they revisit the PEC with every unclear or worsening symptom [15]. Most of our patients were insured, which was consistent with other studies that reported that insured patients had higher revisit rates to PECs than uninsured (self-pay) patients [30,31]. Health insurance offers financial protection against high medical costs; therefore, insured patients have little financial burden when seeking medical care than uninsured patients, who may have to wait until it is necessary to revisit the EC.

Respiratory and gastrointestinal tract infections were the most frequent diagnoses at both the index visit and revisit. Compared with the index visit, the number of gastrointestinal tract, skin, genitourinary and blood infections increased. Other studies reported respiratory, digestive and genitourinary tract infections were the most common diagnoses during revisits [15,30]. In Kenya, malaria, pneumonia, diarrhoea and dehydration were the leading causes of hospital admissions among paediatric patients [32]. In our study, about one in 10 patients with URVs were allergic to drugs or food and 21% had a chronic or pre-existing disease. Asthma, a common risk factor for revisits, was the most prevalent chronic disease among included patients [33]. In Saudi Arabia, paediatric patients with chronic diseases had a URV rate of 11%, with 6.9% returning more than once within 72 hours [30]. Despite the high URV rate among those with chronic diseases in that study, we could not assess the relationship between allergies and chronic diseases with URVs in our study.

We found that the number of urgent and non-ambulatory cases increased between the index visit and the revisit. This increase may be attributable to disease-related factors associated with revisits, such as the natural progression of illnesses that weakens patients and limits their mobility, or chronic diseases, which affected one-fifth of our patients [11]. Also, in Singapore, paediatric patients who arrived by ambulance and were semi-urgent cases were more likely to return [7]. Our study had fewer urgent and semi-urgent cases than most previous studies [7,13,34]. This difference could be explained by differences in the classification of urgency/priority, as well as in the type of patients seen at the PEC. We also found that the number of laboratory and radiological investigations increased between the index visit and revisit. Receiving diagnostic tests during the initial visit has been associated with return to the PEC [16,27]. In our study, the observed increase in diagnostic tests could be attributed to the need to diagnose patients more accurately, but could also highlight potential gaps in the initial patient management.

In our study, most patients were discharged home after review in the PEC. However, about one-fifth were admitted for further management, similar to the rates reported in Canada (19%–21%) [28,29], but lower than that in Singapore (42.8%) [15]. The need for further management could be due to the deterioration of the illness, poor response to initial medications or deficiencies in the initial management of patients in the EC [10]. However, the claim that the reason for URVs could be issues related to the care provided during the first visit has been refuted, as most revisits are related to non-medical reasons [35]. Most patients in our study were discharged home; many were non-urgent or semi-urgent cases. A previous study found that patients who revisited the EC were not more seriously sick than those who had not been seen previously [15,36]. These unnecessary return visits increase demand on the PEC and may hamper service delivery to other patients that need care. To address this, predictive models have been developed to alert health providers of the likelihood of a patient’s revisit [37].

### Strengths and limitations

To our knowledge, this is the first study to characterise paediatric patients who revisited the PEC within 72 hours in Kenya. However, the study findings should be interpreted with caution. First, this study used a retrospective design that limited the amount of data that could be collected because of missing records. For example, we could not collect details of the quality of care provided, discharge instructions provided, interaction time with healthcare providers and length of stay in the PEC, as this information was missing or inconsistently recorded. Second, only paediatric patients aged ≤15 years were included because patients >15 years are seen at the adult EC. Third, this study was conducted among paediatric patients at a single private tertiary referral hospital, which limits the generalisability of the study findings. Fourth, we examined the relationship between the index visit diagnosis and revisit diagnosis, but revisits might have been unrelated to the initial visit.

## Conclusion

The rate of URVs is low in our setting. Paediatric patients who return to the PEC within 72 hours tend to be male, under-5 years, insured, classified as non-urgent cases and have a diagnosis of respiratory or gastrointestinal tract infections. About one-fifth of the patients who revisited were admitted for further management, with admission significantly associated with patient acuity, ambulatory status and laboratory tests. The study findings show that some URVs were necessary and may have contributed to better care and improved health outcomes. There is need, however, for increased emphasis on effective patient education and a comprehensive initial assessment devoid of deficiencies to reduce URVs. Further prospective and qualitative studies are recommended to determine the rate of URVs in public hospitals, explore reasons for URVs and develop models to predict potential return visits in real-time.

## Dissemination of results

The study's results were disseminated to the hospital staff where the data was collected during the weekly paediatrics departmental meeting.

## CRediT authorship contribution statement

Authors contributed as follow to the conception or design of the work; the acquisition, analysis, or interpretation of data for the work; and drafting the work or revising it critically for important intellectual content: KMR contributed 35%; SMG 35%; HMM 15% and RWK contributed 15%. All authors approved the version to be published and agreed to be accountable for all aspects of the work.

## Declaration of competing interest

The authors declared no conflicts of interest.

## References

[1] Di Giuseppe G., Abbate R., Albano L., Marinelli P., Angelillo I.F., Group CR (2008). Characteristics of patients returning to emergency departments in Naples, Italy. BMC Health Serv Res.

[2] Timm N.L., Ho M.L., Luria J.W. (2008). Pediatric emergency department overcrowding and impact on patient flow outcomes. Acad Emerg Med.

[3] Verelst S., Pierloot S., Desruelles D., Gillet J.-B., Bergs J. (2014). Short-term unscheduled return visits of adult patients to the emergency department. J Emerg Med.

[4] Jenab Y., Haghani S., Jalali A., Darabi F. (2015). Unscheduled return visits and leaving the chest pain unit against medical advice. Iran Red Crescent Med J.

[5] Sung S.F., Liu K.E., Chen S.C., Lo C.L., Lin K.C., Hu Y.H. (2015). Predicting factors and risk stratification for return visits to the emergency department within 72 hours in pediatric patients. Pediatr Emerg Care.

[6] Wang K.-C., Chaou C.-H., Liu P.-H., Chien C.-Y., Lee C.-H. (2017). Factors affecting unscheduled return visits to the emergency department among minor head injury patients. Biomed Res Int.

[7] Chan A.H., Ho S.F., Fook-Chong S.M., Lian S.W., Liu N., Ong M.E. (2016). Characteristics of patients who made a return visit within 72 hours to the emergency department of a Singapore tertiary hospital. Singapore Med J.

[8] Easter J.S., Bachur R. (2013). Physicians’ assessment of pediatric returns to the Emergency Department. J Emerg Med.

[9] KilicaSlan O., Sönmez F.T., Gunes H., Temizkan R.C., Kocabay K., Saritas A. (2017). Short term unscheduled revisits to paediatric emergency department-a six year data. J Clin Diagn Res.

[10] Akenroye A.T., Thurm C.W., Neuman M.I., Alpern E.R., Srivastava G., Spencer S.P. (2014). Prevalence and predictors of return visits to pediatric emergency departments. J Hosp Med.

[11] Bernstein S.L., Aronsky D., Duseja R., Epstein S., Handel D., Hwang U. (2009). The effect of emergency department crowding on clinically oriented outcomes. Acad Emerg Med.

[12] Zed P.J., Abu-Laban R.B., Balen R.M., Loewen P.S., Hohl C.M., Brubacher J.R. (2008). Incidence, severity and preventability of medication-related visits to the emergency department: a prospective study. Can Med Assoc J.

[13] Vijay J., Mathew N., Guttikonda A., Mitra S., Abhilash K.P. (2020). Emergency department revisits within 72 hours to a tertiary care referral hospital in south India. Current Medical Issues.

[14] Burokiene S, Kairiene I, Stricka M, Labanauskas L, Cerkauskiene R, Raistenskis J, et al. Unscheduled return visits to a pediatric emergency department. Medicina (Kaunas). 2017;53:66-71. doi:10.1016/j.medici.2017.01.003. 10.1016/j.medici.2017.01.003 28233682

[15] Goh GL, Huang P, Kong MC, Chew SP, Ganapathy S. Unplanned reattendances at the paediatric emergency department within 72 hours: a one-year experience in KKH. Singapore Med J. 2016;57:307-13. doi:10.11622/smedj.2016105. 10.11622/smedj.2016105 PMC4971449 27353384

[16] Goldman R.D., Ong M., Macpherson A. (2006). Unscheduled return visits to the pediatric emergency department-one-year experience. Pediatr Emerg Care.

[17] Barnes J, O'Hanlon B, Feeley FI, McKeon K, Gitonga N, Decker C. Private Health Sector Assessment in Kenya. World Bank Working Paper. The World Bank; 2010.

[18] Obermeyer C.M., Baijal P., Pegurri E. (2011). Facilitating HIV disclosure across diverse settings: a review. Am J Public Health.

[19] Ministry of Health (2014). 2013 Kenya Household Health Expenditure and Utilisation Survey.

[20] Kenya National Bureau of Statistics (2020). Economic Survey 2020.

[21] Ilinca S., Di Giorgio L., Salari P., Chuma J. (2019). Socioeconomic inequality and inequity in use of health care services in Kenya: evidence from the fourth Kenya household health expenditure and utilization survey. Int J Equity Health.

[22] Ministry of Health. Kenya Health Policy 2015 - 2030. Nairobi, Kenya: Ministry of Health; 2015.

[23] Ministry of Health (2020). Kenya Harmonized Health Facility Assessment (KHFA), 2018/2019.

[24] Kazungu J.S., Barasa E.W. (2017). Examining levels, distribution and correlates of health insurance coverage in Kenya. Trop Med Int Health.

[25] Warren D.W., Jarvis A., LeBlanc L., Gravel J., Group CNW (2008). Revisions to the Canadian triage and acuity scale paediatric guidelines (PaedCTAS). Canadian Journal of Emergency Medicine.

[26] Reiser O., Diamand R., Shavit I. (2019). Early unplanned return visits to a pediatric emergency department in Israel. Pediatr Int.

[27] Hu Y.H., Tai C.T., Chen S.C., Lee H.W., Sung S.F. (2017). Predicting return visits to the emergency department for pediatric patients: applying supervised learning techniques to the Taiwan National Health Insurance Research Database. Comput Methods Programs Biomed.

[28] Logue E.P., Ali S., Spiers J., Newton A.S., Lander J.A. (2013). Characteristics of patients and families who make early return visits to the pediatric emergency department. Open Access Emerg Med.

[29] Goldman R.D., Kapoor A., Mehta S. (2011). Children admitted to the hospital after returning to the emergency department within 72 hours. Pediatr Emerg Care.

[30] Ahmed A.E., Bi A.L., Alrajhi M.N., Almazroa H.R., DA AlBuraikan, Albaijan M.A. (2018). Emergency department 72-hour revisits among children with chronic diseases: a Saudi Arabian study. BMC Pediatr.

[31] Alpern E.R., Clark A.E., Alessandrini E.A., Gorelick M.H., Kittick M., Stanley R.M. (2014). Recurrent and High-frequency use of the emergency department by pediatric patients. Acad Emerg Med.

[32] Ayieko P., Ogero M., Makone B., Julius T., Mbevi G., Nyachiro W. (2016). Characteristics of admissions and variations in the use of basic investigations, treatments and outcomes in Kenyan hospitals within a new Clinical Information Network. Arch Dis Child.

[33] Quincy Khoi Tran, Jamil D. Bayram, Romsai T. Boonyasai, Meredith A. Case, Christine Connor, David Doggett, et al. Pediatric emergency department return: a literature review of risk factors and interventions. Pediatr Emerg Care. 2016;32. doi:10.1097/PEC.0000000000000876. 10.1097/PEC.0000000000000876 27490736

[34] de Vos-Kerkhof E., Geurts D.H.F., Steyerberg E.W., Lakhanpaul M., Moll H.A., Oostenbrink R. (2018). Characteristics of revisits of children at risk for serious infections in pediatric emergency care. Eur J Pediatr.

[35] Ali A.B., Place R., Howell J., Malubay S.M. (2012). Early pediatric emergency department return visits: a prospective patient-centric assessment. Clin Pediatr (Phila).

[36] Pham J.C., Kirsch T.D., Hill P.M., DeRuggerio K., Hoffmann B. (2011). Seventy-two-hour returns may not be a good indicator of safety in the emergency department: a national study. Acad Emerg Med.

[37] Jin B., Zhao Y., Hao S., Shin A.Y., Wang Y., Zhu C. (2016). Prospective stratification of patients at risk for emergency department revisit: resource utilization and population management strategy implications. BMC Emerg Med.

